# Jejunal pouch reconstruction after total gastrectomy is associated with better short-term absorption capacity and quality of life in early-stage gastric cancer patients

**DOI:** 10.1186/s12893-018-0397-0

**Published:** 2018-08-20

**Authors:** Wei Chen, Mianxu Jiang, Hui Huang, Zao Ding, Chihua Li

**Affiliations:** 1 Department of Gastrointestinal Surgery, The Central Hospital of the Wuhan, Tongji Medical College, Huazhong University of Science and Technology, Wuhan, 430000 People’s Republic of China; 2 Department of General Surgery, Zhongnan Hospital of Wuhan University, Wuhan, 430014 People’s Republic of China; 3 Department of Gastrointestinal Surgery, Hubei Cancer Hospital, Wuhan, 430014 People’s Republic of China

**Keywords:** Total gastrectomy, Reconstruction, Jejunal pouch, Quality of life

## Abstract

**Background:**

No consensus exists regarding the best reconstruction style after total gastrectomy (TG). Roux-en-Y oesophagojejunostomy is a simple option for gastrointestinal tract reconstruction. Recently, jejunal pouch reconstruction has been suggested as an appropriate approach. We compared the postoperative outcomes of the two surgical approaches using a well-characterized cohort of gastric carcinoma patients.

**Methods:**

A total of 60 patients who underwent TG were divided into two groups according to the reconstruction style. Both groups were compared regarding patient characteristics, perioperative data and quality of life (QoL), which was assessed using the Spitzer QoL index (QLI) and Visick grade. The incidence of long-term surgery-related complications, including reflux oesophagitis, dumping syndrome, and retention syndrome, was also compared to evaluate postoperative restoration.

**Results:**

Both study groups were comparable with respect to general patient characteristics. No mortality or no significant differences in surgery-related data were found except in the operation time. Compared to Orr Roux-en-Y reconstruction, pouch reconstruction was associated with a longer procedure time, a lower incidence of dumping/retention syndrome and better QoL parameters (*p* < 0.05).

**Conclusion:**

In this study, jejunal pouch reconstruction after TG was superior to the traditional Roux-n-Y oesophagojejunostomy with respect to improved dietary intake and QoL.

## Background

Gastric cancer is a common gastrointestinal (GI) tract malignant tumour disorder with high morbidity and mortality [1], and surgical intervention remains a cornerstone for treating gastric cancer. Radical total gastrectomy (TG) is one of the common procedures of choice and has provided relief for stomach cancer via wide use over more than 100 years [2]. Appropriate GI reconstruction is most likely associated with a better quality of life (QoL) [3], and various styles of GI reconstruction can be applied after TG. The preferred approach for reconstruction of the digestive tract following total stomach resection is oesophagojejunostomy with pouch formation and duodenal transit preservation [4]. However, the procedure is complex and difficult to promote in basic-level hospitals in China. In our hospital,pure jejunal pouch reconstruction with Roux-en-Y oesophagojejunostomy has been shown to be an alternative approach as it provides a reservoir for digestion and absorption and is easy to brand. However, studies in the literature describing this technique are scarce.The aim of the present study was to investigate the operation-related complication rate, nutritional status, prognosis and quality of daily life following TG with jejunal pouch reconstruction in three tertiary institutions. For this purpose, we have used defined and validated scoring systems to evaluate postoperative functional outcomes, as previously reported [5–7].

## Methods

### Reconstruction technique

**Fig. 1:**
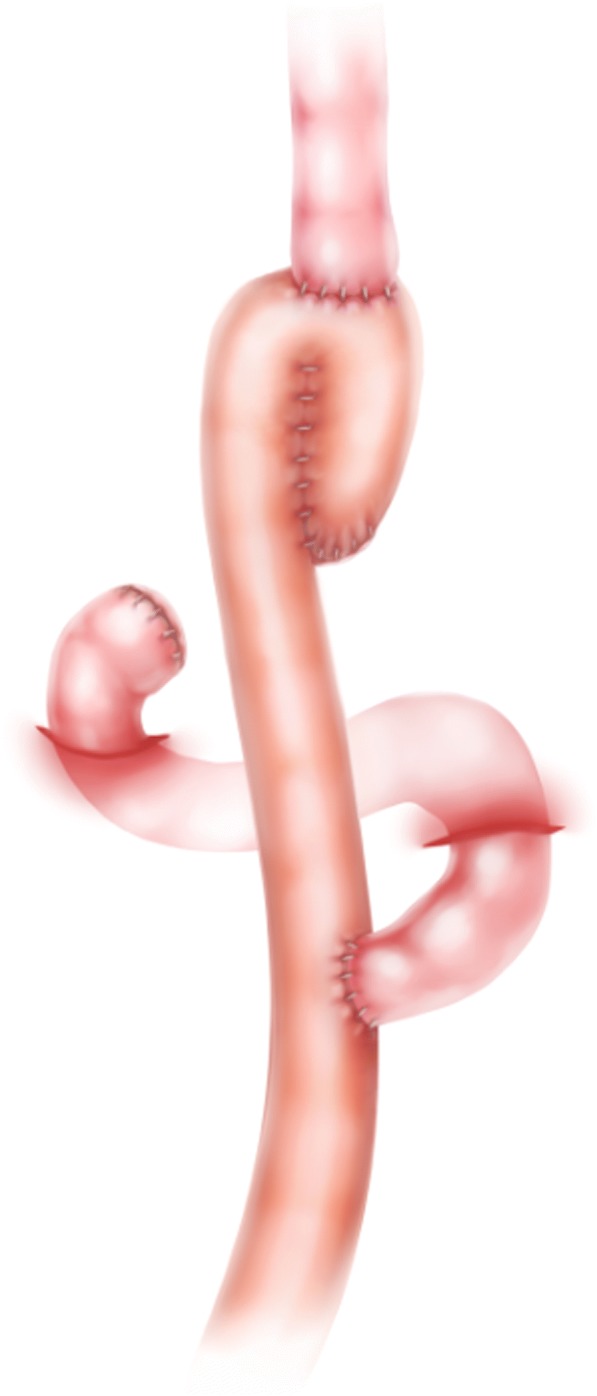
Schematic illustration of the jejunal pouch after total gastrectomy, which was accomplished using a linearstapler: the jejunum was repositioned to allow anastomosis(using a 100-mm linear stapler) with no tension and a larger capacity

**Fig. 2:**
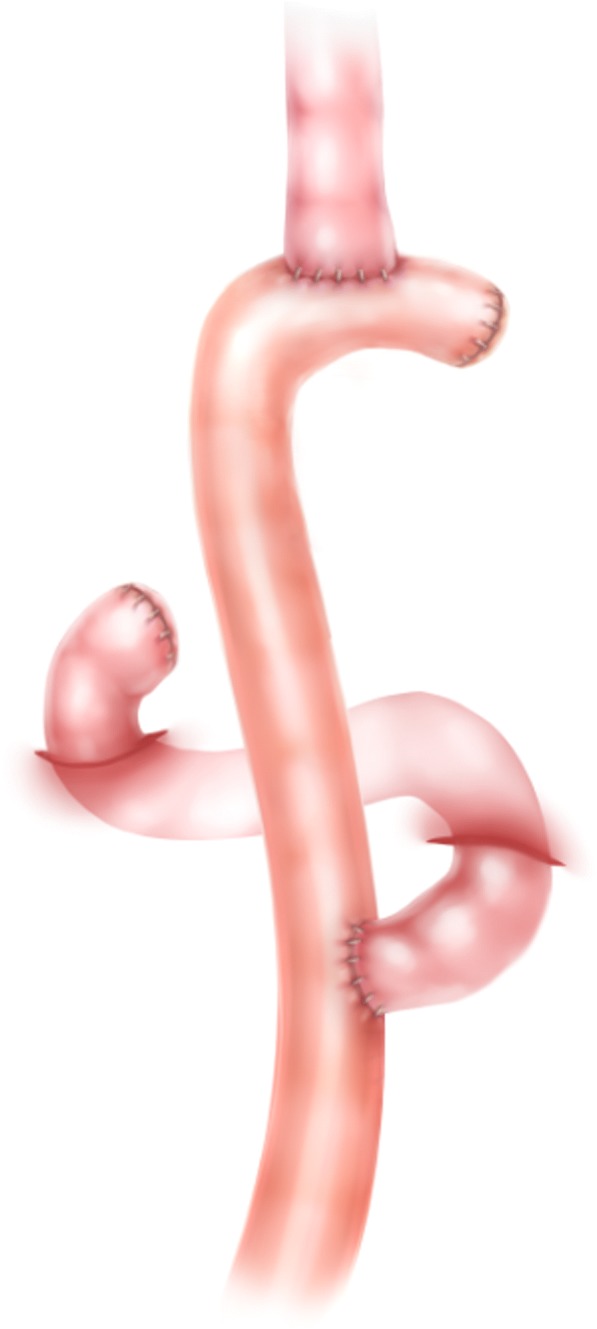
Schematic illustration of the conventional OrrRoux-en-Y technique. Aftertotal gastrectomy, this type of reconstruction was performed using the double-staplingtechnique

In 1952, Hunt and Cope [8, 9] first reported a pouch or reservoir fashioned from a loop of the jejunum with the Roux-en-Y principle of oesophagojejunostomy. Ten years later, Lawrence [10] modified this procedure and successfully used it in several patients after gastrectomy. Here, we present a modified pouch reconstruction style using stapled anastomosis, which is safe and convenient.Abdominal radical gastrectomy for cancer was performed according to the routine procedure [11].After removal of the entire stomach, the ligament of Treitz was identified, and a pre-removal line approximately 30–40 cm from the ligament was marked. A loop of the jejunal bowel that was freely mobile was selected and divided before bringing the already divided distal jejunum up to the lower oesophagus to complete the future end for the pouch reconstruction. Before this key step, the divided distal jejunum was folded on itself in a form similar to a reservoir; pouch reconstruction was primarily performed using a 100-mm linear stapler(Johnson linear stapler, TLC100 Proximate,USA),which provides the largest possible scale (Fig. 1). After creation of the jejunal pouch, the oesophagojejunostomy was finished using a circular stapler (Johnson columnar stapler, CDH25A/29A,USA)with the anvil inserted through the oesophagus and the stapler inserted through the enterotomy of the pouch. Then, the enterotomy was closed with 3–0 polydioxanone (PDS, Ethicon, Cincinnati, USA).Notably,after the anvil was inserted into the oesophageal stub, a purse-string suture was placed to secure it, preventing retraction of the oesophageal mucosa and increasing the possibility of obtaining complete loops during the anastomotic process. Next, the Roux-Y jejuno-jejunostomy was created viaside-to-side hand-sewn anastomosis 2 cm in diameter with continuous 3–0 PDS, approximately 50 cm from the site of the future oesophageal-jejunal anastomosis (EJA). For the conventional Roux-en-Y procedure, EJA was performed with a simple end-to-side technique (Fig. 2). Because all patients underwent typical abdominal TG with different digestive reconstruction methods, the surgical outcomes are sufficiently comparable for comparison.

### Patients

**Fig. 3:**
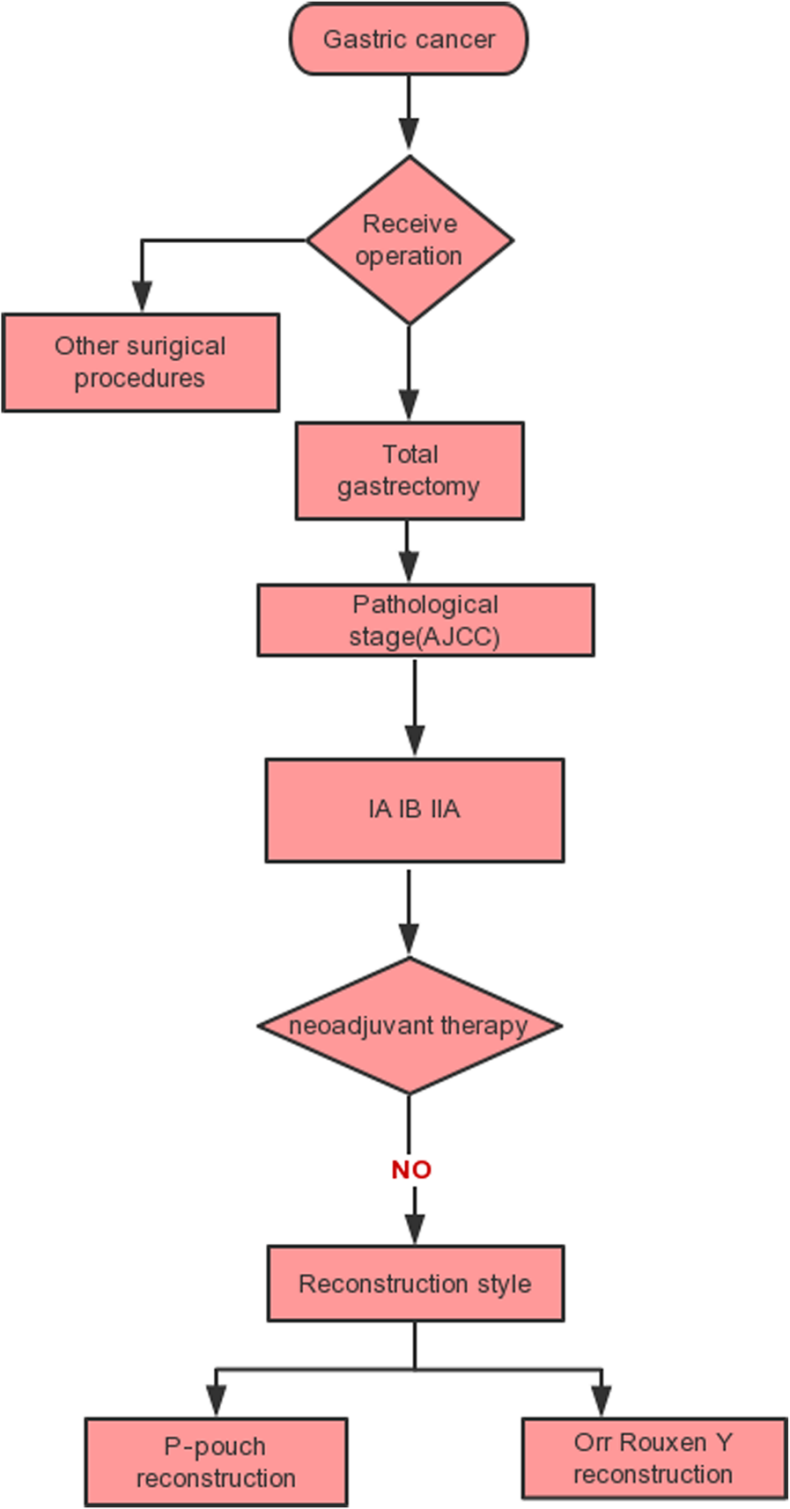
A flowchart of the selection of patients involved in this study

Sixty patients who underwent abdominal TG with jejunal pouch reconstruction or simple Roux-en-Y anastomosis at three tertiary hospitals(the Central Hospital of Wuhan/Zhongnan Hospital of Wuhan University/Hubei Cancer Hospital) between January 2010 and December 2015 were reviewed. The clinicopathological data are shown in Table 1. Thirty-two patients underwent TG with jejunal pouch reconstruction, whileanother twenty-eight patients underwent single Roux-en-Y reconstruction after TG. All preoperative and postoperative data were reviewed using the institutional surgical databases involved in this research. The inclusion and exclusion criteria for patient selection are shown in a simple flow chart (Fig. 3).

**Table 1:** Patients’ general information

Clinicopathologic feature	P-pouch group(*n* = 32)	Orr-RYgroup (*n* = 28)	P
Age (<60y/≥60y)	12/20	10/18	0.886
Sexual (male/female)	21/9	18/10	0.643
Pathology grading			0.499
Well differentiated	12	7	
Moderate differentiated	19	19	
Poor differentiated	1	2	
TNM			0.577
IA	7	4	
IB	19	16	
IIA	6	8	
BMI	23.90 ± 2.3	23.2 ± 2.1	0.153
ALB	44.6 ± 6.2	42.9 ± 5.3	0.254
HB	124.4 ± 10.2	120.8 ± 8.3	0.137

### Functional outcome assessment

To evaluate postoperative recovery, two validated and internationally accepted tools were employed in this research for data collection: the modified Spitzer quality of life index (QLI) and the Visick grade [12, 13]. These assessment tools are considered reliable and objective methods for assessing the outcomes of patients after GI surgery. The modified Spitzer QLI focuses on oral food intake and its influence on daily life, and the index ranges from 1 (poor) to 3 (fine).The maximum score is 52,with higher scores representing better outcomes. The Visick grade ranges from 1 (excellent) to 4 (poor) and focuses on disease-related mental states and relevant digestive tract symptoms. In general, lower Visick grades and higher Spitzer QLIs represent better postoperative recovery. Moreover, the prognostic nutritional index (PNI) was also used to evaluate the nutritional condition of patients in this research. The PNI was calculated according to the serum albumin concentration and peripheral blood lymphocyte count with the aim of evaluating the preoperative nutritional status, the risk of surgical infection and postoperative complications in the patients; the PNI is currently widely used in patients after GI and cardiac surgery [14].All assessments were carried out at three months, six months and one year after the operation. Data were collected through the outpatient clinic as well as through a telephone interview.

### Statistical analyses

Variables that fulfilled the criteria for a normal distribution were analysed using the Kolmogorov–Smirnov test.Normally distributed data are expressed as the mean ± SD and were analysed by two-tailed Student’s t tests.The chi-square test was used to assess categorical data. Statistical analyses were performed using SPSS software, version 19.0(SPSS, Chicago, IL, USA).A difference between groups with a *p*-value of < 0.05 was considered statistically significant.

## Results

**Fig. 4:**
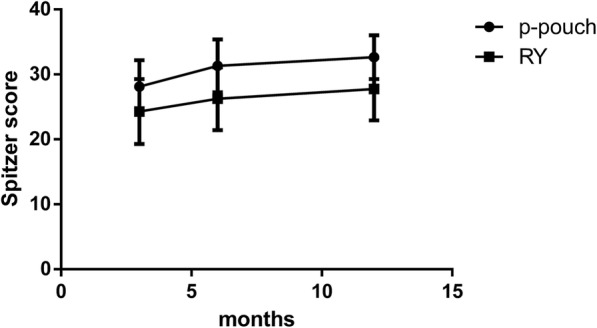
The Spitzerquality of life index (QLI)at different times after the two surgical procedures. Data collected from the first, second and third follow-up visits showed a higher QLI (represented by the mean ± SD) in the jejunalpouch group than in the Orr Roux-en-Y group (*p* < 0.05)

**Fig. 5:**
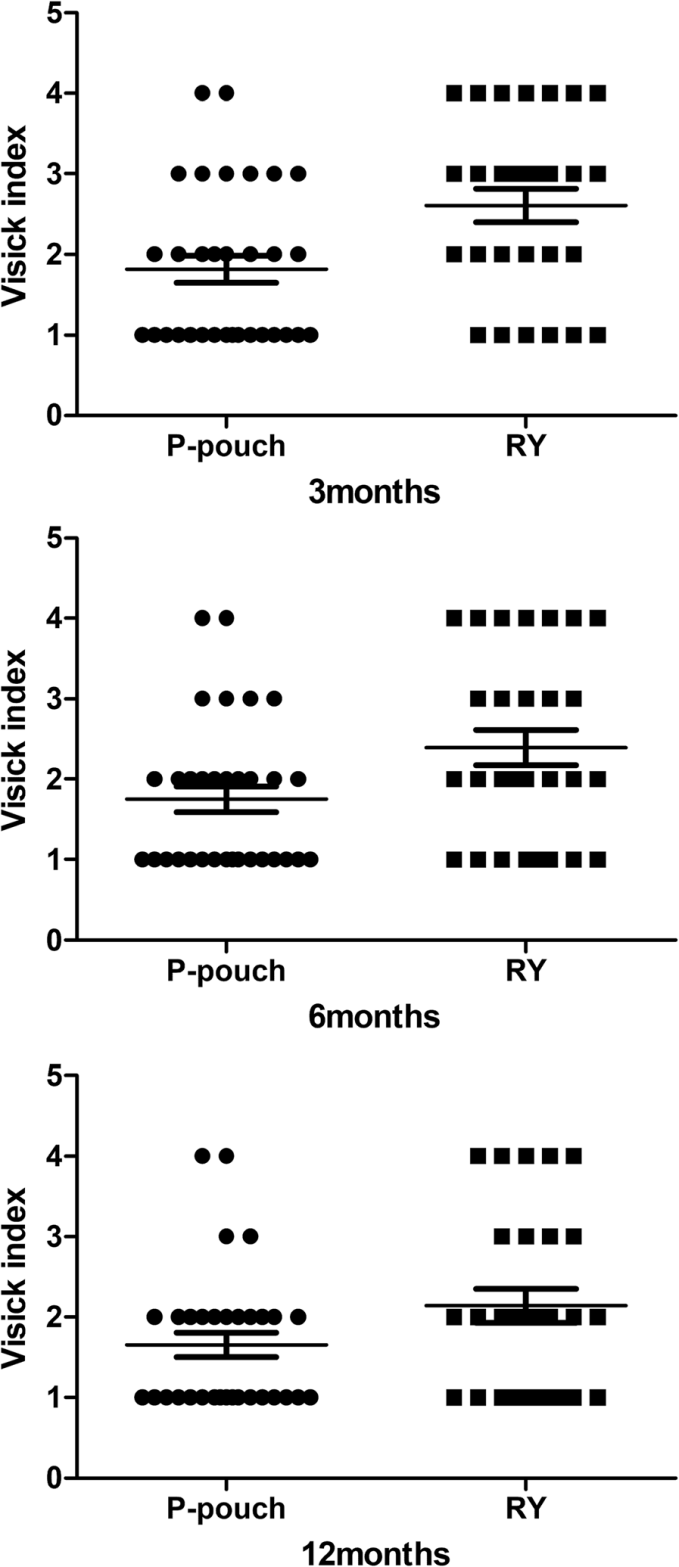
The Visick grade at different times afterthe two surgical procedures. Data collected from the first two follow-up visits showed better performance (represented by a ratio) in the jejunalpouch group than in the Orr Roux-en-Y group (*p* < 0.05). The last follow-up visit showed no significant difference between the 2 groups (*p* > 0.05)

**Fig. 6:**
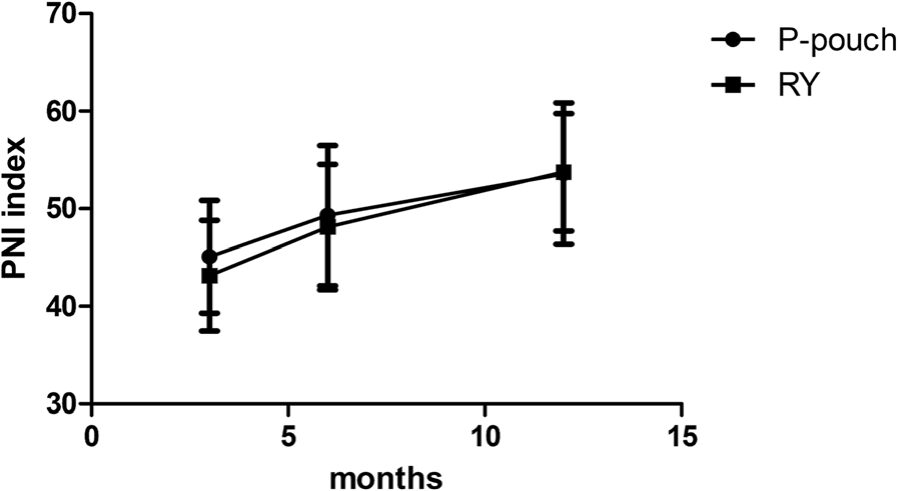
The prognostic nutritional index (PNI) performed better in the jejunal pouch group; however, the difference was notsignificant (*p* > 0.05)

No significant differences were found between the groups in demographic or clinical characteristics (Table 1). We considered the cohort suitable for comparing the outcomes of jejunal pouch reconstruction and simple Roux-en-Y. The surgery-related results and the patient satisfaction rate are shown in Table 2. No mortality was found during the early postoperative period. Anastomotic fistulae occurred in five patients after the operation. Anastomotic bleeding or stenosis was not observed. The more common complications directly related to surgery were paralytic ileus (33.3%) and pulmonary infection (31.6%). No significant differences were found between the two digestive tract reconstruction styles in any of the factors that were directly linked to the expected operation time(245.8 ± 27.6 vs. 222.7 ± 24.7,*p* = 0.01).Other severe surgery-related complications, such as pancreatic fistula and abdominal abscess, were not found in our cohort. Patients were generally satisfied with the surgical outcomes, and the vast majority of patients reiterated that under the same circumstances, they would opt for pouch reconstruction again(78.1%).Up to 3 months after the operation, significantly less abdominal discomfort, appetite loss and weight loss were observed in the jejunal pouch group than in the conventional Orr-RY group(3-month Spitzer QLI:28.1 ± 4.0 vs. 24.3 ± 4.9, *p* = 0.002).Similar results were observed again during the follow-up period (6-month Spitzer QLI:31.3 ± 4.0 vs.26.3 ± 4.8, *p* = 0.000; 12-monthSpitzer QLI: 32.7 ± 3.4 vs. 27.8 ± 4.8, *p* = 0.000). Interestingly, both sets of data related to postoperative rehabilitation improved over time in these patients, probably due to general improvements in diet control and the adaptive capacity of body over the long term (Fig. 4). Similar outcomes were observed for the Visick grade in the two groups (Fig. 5). At the first two follow-up time points, a discernible difference between the jejunal pouch and Orr Roux-en-Y reconstruction groups was identified by univariate analysis (I/IIvs III/IV at 3 months:*p* = 0.005; I/IIvsIII/IV at 6 months: *p* = 0.028).However, at the last follow-up time point, the jejunal pouch procedure was no longer superior to Orr Roux-en-Y with respect to the Visick grade (I/IIIvsIII/IV at 12 months: *p* = 0.137).Regarding prognostic indicators, the PNI was better in the jejunal pouch group, although the difference was not significant (Fig. 6).To assess long-term complications, we conducted a survey 1 year after the operation.The incidence of reflux oesophagitis(RE)was rare in the jejunal pouch group compared to that in the traditional OrrRoux-en-Y group, although the difference was not significant. Complaints about dumping syndrome and retention syndrome were significantly more commonly reported in the conventional OrrRoux-en-Y group during the follow-up phone calls (Table 3).

**Table 2:** Surgical results

Parameters	P-pouch group(*n* = 32)	Orr-RY group(*n* = 28)	P
Operative time(min)^a^	245.8 ± 27.6	222.7 ± 24.7	0.010
Blood loss in operation(ml)	301.3 ± 80.9	283.2 ± 60.7	0.339
Iincision infection	2/32	1/28	0.635
Small bowel obstruction	9/32	11/28	0.360
Pulmonary infection	10/32	9/28	0.941
Anastomotic fistula	3/32	2/28	0.755
Hospital stay(day)	12.6 ± 1.6	12.3 ± 1.7	0.589
Satisfaction rate	25/32	20/28	0.550

^a^Significant difference bewteen the P-pouch and Orr-RY group parameters

**Table 3:** Long term complication comparison 12 months after operation

Complication	P-pouch group(*n* = 32)		Orr-RY group(*n* = 28)		P
	Case number	ratio%	Case number	ratio%	
RE syndrome	11	34.3	16	57.1	0.006
Dumping syndrome^a^	2	6.2	8	21.0	0.021
Retention syndrome^a^	3	9.3	10	35.7	0.013

*RE* reflux esophagitis.^a^Significant difference bewteen the P-pouch and Orr-RY group parameters

## Discussion

In 1897, George Schlatter performed TG in a female patient with gastric cancer in Zurich using simple EJA to rebuild the digestive tract;however,anaemia and diarrhoea were noticeable and insurmountable [15].Patients who undergo TG usually complain of diarrhoea, upper abdominal pain after dinner, RE, early or late dumping syndrome and refractory anaemia, collectively called postgastrectomy syndrome [16]. To resolve this outcome, continuous improvement in the reconstruction of the alimentary tract after TG has occurred over the past 100 years; more than 50 reconstruction types have been reported in the literature [2, 15].Unfortunately, no gold standard has been established due to a lack of clinical evidence. In China,the commonly used strategy is called the modified Orr Roux-en-Y style [17].This reconstruction method has the advantage of being easy to perform, less traumatic and associated with reduced surgery-related morbidity. However, a relatively higher incidence of bowel-related complications is a disadvantage of this technique due to the lack of physiological storage and rapid food emptying [18].

The currently increasing emphasis on the recovery of psychological and social parameters, coupled with changes in health conception and medical models, has drawn our attention.Both the Spitzer QLI questionnaire and Visick grade self-assessment form are widely used for assessing the QoL of GI tumour patients after surgery.Reconstruction of the GI tract not only involves a continuation of the anatomical structure but also aims to preserve as much of the physiological function as possible. Choosing an appropriate reconstruction method is intrinsic to achieving satisfactory postoperative outcomes, reducing complication rates and improving the QoL.

Little is known about the efficacy of jejunal pouch reconstruction after TG [19].It has been suggested that TG with Roux-en-Y reconstruction might lead to a poor QoL due to malnutrition and intractable dumping syndrome. Recently, several clinical studies have reported that the presence of a reservoir after TG is related to an increased body weight and better QoL [20–22].In contrast, Fujiwara Y demonstrated that the benefit of constructing are servoir after TG is limited [23].Miyoshi K also argued that pouch reconstruction after TG does not significantly contribute to weight gain [24].Accordingly, the desirability of reservoir reconstruction after TG remains unclear.

In this study, after analysing the postoperative follow-up data, we found that patients who underwent reservoir reconstruction showed an improved body mass and daily QoL, which may support the former view point. In general, both traditional OrrRoux-en-Y and jejunal pouch reconstruction strategies appear to work well. No surgery-related mortality occurred during our study. Furthermore, no significant differences were found in terms of the clinical data or surgery-related complications.However, it is worth noting that the operation time in the jejunal pouch group was longer than that in the OrrRoux-en-Y group, but the extended operation time did not seem to be harmful. This factor served to minimize the chances of any spurious differences influencing the long-term results, and it truly increased the ascription of the success(or otherwise) of the reconstruction style to the intrinsic properties of the particular surgical technique. Moreover, with the greater application of the reconstruction technique, surgeons will become more proficient, and the operation times will become shorter. In recent years, with the application of stapling, the reconstruction procedure has become rapid, safe and practical [25].With respect to postoperative reflux symptoms or related morbidities, although no significant difference was found during the follow-up period,the lower postoperative reflux rate in the jejunal pouch group was consistent with the notion that pouch reconstruction achieves better results. Compared to traditional reconstruction, this reconstruction resulted in a lower incidence of dumping syndrome and/or retention syndrome, better postoperative recovery and a better global health status.A noteworthy observation was the general occurrence of gastrointestinal dynamic disorders, nutrient deficiencies and anaemia during the initial postoperative period,which often necessitated pharmacological intervention due to absence of the stomach.However, for the majority of the patients, these symptoms eventually resolved over time.As documented during the follow-up examinations, the incidence of surgery-related complications, including RE,dumping syndrome,diarrhoea and retention syndrome, after EJA with Roux-en-Y reconstruction or pouch-style reconstruction can be controlled, as improvements would achieved in both two groups.This finding is in line with the results reported by Iivonen MK [26] andis possibly related to the adaptation capacity of the body and diet control. In their study, postoperative dumping and early satiety were more common in the Roux-en-Y group after 3 months. In the jejunal pouch group, better results were found in terms of intestinal motility and the nutritional status, demonstrating that the effect of the jejunal pouch reconstruction technique on GI symptoms was more pronounced than that of the traditional OrrRoux-en-Y reconstruction technique. Bracale U reported a lower rate of anastomosis leaks and more comfortable deglutition achieved with SS-stapled anastomosis after oesophagectomy [27]; these results are consistent with the gastrectomy results presented here.

In our opinion, jejunal pouch reconstruction has several advantages. It may alleviate postoperative gastric-related complications because the resulting curvature has valve-like characteristics that mitigate reflux disease and effectively change the direction of food,thereby giving the chyme more time to be assimilated.Additionally, this reconstruction style is not restricted by the intestinal diameter, thereby reducing the risk of strictures. Moreover, the OrrRoux-en-Y procedure produces a distal closed loop of jejunal bowel near the anastomotic site, easily leading to pouchitis. Thus, the pouch technique appears to be a more physiological reconstruction method for application in patients undergoing TG.

There were certain limitations in our research. First, the short follow-up period—just 12 months—was a limitation, and the data analysed were inferior to data more indicative of the dynamic state; additionally, the sample size was small. Second, a relatively simple questionnaire was involved in the data analysis, and other parameters might also influence the analysed outcomes. Clearly, the perceptions of the definition of functional recovery may also lead to an unconscious bias among participants completing the modified Spitzer QLI questionnaire and the Visick grade self-assessment form; thus, the data rely on the individual understanding of each patient. Finally, the exact mechanism mediating the reduced incidence of dumping syndrome and retention syndrome and the improved daily QoL after pouch reconstruction was not clear. Regarding the latter, the morphological similarity of the reconstruction might play a critical role in this process.

## Conclusion

In summary,TG with jejunal pouch reconstruction for gastric cancer is feasible and safe.It combines the advantages of improved digestive absorption and storage and improved QoL. Based on our results, jejunal pouch reconstruction appears to be a superior surgical approach. However, more randomized controlled clinical studies are essential to verify the benefits of this procedure.

## Abbreviations

EJA: Oesophageal-jejunalanastomosis
PNI: Prognostic nutritional index
QoL: Quality of life
RE: Reflux oesophagitis
TG: Total gastrectomy
