# Correction to: Jejunal pouch reconstruction after total gastrectomy is associated with better short-term absorption capacity and quality of life in early-stage gastric cancer patients

**DOI:** 10.1186/s12893-018-0403-6

**Published:** 2018-09-13

**Authors:** Wei Chen, Xumian Jiang, Hui Huang, Zao Ding, Chihua Li

**Affiliations:** 1 Department of Gastrointestinal Surgery, The Central Hospital of the Wuhan, Tongji Medical College, Huazhong University of Science and Technology, Wuhan, 430000 People’s Republic of China; 2 Department of General Surgery, Zhongnan Hospital of Wuhan University, Wuhan, 430014 People’s Republic of China; 3 Department of Gastrointestinal Surgery, Hubei Cancer Hospital, Wuhan, 430014 People’s Republic of China

## Correction

Following publication of the original article [1], the authors reported that one of the authors’ names is spelled incorrectly. In this Correction the incorrect and correct author name are shown. The original publication of this article has been corrected.

Originally the author name has been published as:

- Mianxu Jiang

The correct author name is:

- Xumian Jiang
